# Associations between Active Mobility Index and objectively measured physical activity among older adults

**DOI:** 10.1007/s40520-022-02256-z

**Published:** 2022-10-21

**Authors:** Satoshi Kurita, Takehiko Doi, Kota Tsutsumimoto, Sho Nakakubo, Yuto Kiuchi, Kazuhei Nishimoto, Hiroyuki Shimada

**Affiliations:** 1 Department of Preventive Gerontology, Center for Gerontology and Social Science, Research Institute, National Center for Geriatrics and Gerontology, 7-430 Morioka-choAichi, Obu, 474-8511 Japan; 2 Graduate School of Health Sciences, Kagoshima University, Kagoshima, Japan; 3 Department of Medical Sciences, Medical Science Division, Graduate School of Medicine, Science and Technology, Shinshu University, Nagano, Japan

**Keywords:** Life space, Outdoor activity, Physical activity intensity

## Abstract

**Background:**

Active mobility index (AMI) is a questionnaire to assess going-out behavior with physical and social activity. The association between AMI scores and objectively measured physical activity (PA) in older adults is unknown.

**Methods:**

Community-dwelling older adults aged ≥ 70 years participated in an examination and wore a triaxial accelerometer for seven or more days. The accelerometer measured the time of moderate-to-vigorous intensity PA (MVPA) and light intensity PA (LPA), and step counts. The AMI assessed life space (distance from the respondent’s home: < 1, 1–10, or > 10 km) and related activities during the previous month. The AMI total, physical, and social scores were calculated.

**Results:**

The analyzed data were 2499 participants (mean age: 75.5 ± 4.0 years; 54.4% female). Comparing PA among quartile groups of each AMI score, higher AMI total and physical score groups were associated with higher MVPA, LPA, and step counts (all *P* < 0.01). The Q4 group of AMI social scores showed significantly higher LPA and step counts than the Q1 and Q2 groups (*P* < 0.01). The logistic regression model showed higher score groups of AMI total and physical scores associated with increased adjusted odds ratio (aOR) of meeting recommended PA, ≥ 150 min/week of MVPA.

**Conclusions:**

Older adults with higher AMI total and physical scores, engaged in more PA. Future studies can use the present findings when estimating PA in older adults from AMI scores and examining the association between AMI scores and health outcomes.

## Introduction

Life space is regarded as an important factor in older adult’s health [1]. Extensive research has reported that life space predicts health outcomes, including cognitive decline, falls, mortality, and quality of life [1]. To assess life space and related activities simultaneously, a previous study developed an active mobility index (AMI) which includes a questionnaire to evaluate life space with physical activity (PA) and social activity [2]. In a longitudinal study, higher AMI total and physical scores were associated with a reduced risk of disability onset [2].

The linkage between life space and health in order adults has been partly explained by PA. Maintenance of PA contributes to the prevention of chronic diseases or functional decline [3–5] and a larger life space is associated with greater engagement in PA [6, 7]. A longitudinal study using an accelerometer found that lower step counts and less time spent in moderate activity at baseline were prospectively associated with a reduced life-space mobility score over 2 years [7]. A cross-sectional study showed that older adults who moved beyond the neighborhood more frequently were inclined to engage in longer moderate-and low-intensity PA [6]. However, the association between AMI and the amount of PA is unknown. To examine the mechanism of the link between AMI and health, knowledge of whether AMI scores are associated with PA is required. The present study aimed to examine the association between AMI scores and objectively measured PA in community-dwelling older adults.

## Methods

### Participants

This cross-sectional observational study used data from the National Center for Geriatrics and Gerontology-Study of Geriatric Syndromes (NCGG-SGS). This community-based cohort study aimed to establish a screening program for geriatric syndromes and to validate evidence-based interventions for their prevention [8]. The eligibility criterion for participation in this study was age ≥ 70 years.

Of the 5257 participants at baseline examination, 5178 agreed to an additional survey and were provided with an accelerometer to measure daily PA. The exclusion criteria in this study were as follows: invalid accelerometer data (*n* = 2260; 1034 participants did not have the data within 30 days after examination, 1208 participants did not wear the accelerometers for 10 h per day for at least 7 days, and 18 participants experienced technical errors with the data reader); the need for support or care certified by LTCI before the examination (*n* = 2); having a self-reported basic activities of daily living (BADL) disability (*n* = 4); having a medical history including stroke, dementia, Parkinson’s disease, or depression (*n* = 302); having a cognitive impairment (mini-mental state examination [MMSE] score < 21 [9] [*n* = 50]); or having missing data (*n* = 61). Finally, a total of 2499 participants were included in the analysis. All participants provided written informed consent before participation. This study was conducted in accordance with the guidelines proposed in the Declaration of Helsinki, and the study protocol was reviewed and approved by the research ethics committee of the National Center for Geriatrics and Gerontology.

### Measurement

#### Physical activity

PA was assessed using a triaxial accelerometer (GT40-020; Kao Corporation, Tokyo, Japan). The display of the accelerometer was set as blinded, and participants were instructed to wear it on either the left or right side of the waist for a month, except during water-based activities, such as bathing or swimming. To collect accelerometer data, the FeliCa RC-S380 data reader (SONY, Tokyo, Japan) was set in nine regional cooperative pharmacies. Participants were instructed to visit these pharmacies and use the data reader within 30 days of the examination. Inclusion criteria for accelerometer data were those who had more than 7 days of data within 30 days after the examination with ≥ 10 h of recording [10] excluding non-wearing time designated as ≥ 35 min of non-recorded time.

The accelerometer estimated the intensity of PA using 11 levels, on a scale of 0.5 (minimal intensity) to 9 (maximal intensity), and recorded the duration in 4-s epoch lengths similar to the Kenz Lifecoder (Suzuken Corporation, Limited: Aichi, Japan) [11]. One to three levels corresponds to ≥ 1.6 to < 3.0 METs, light intensity PA (LPA) (1.6–3 METs), and four or higher levels corresponds to ≥ 3.0 or more METs, moderate-to-vigorous intensity PA (MVPA). The accelerometer also measured step counts. As a secondary outcome, those who met the recommended PA guideline (≥ 150 min/week of MVPA) were identified by referring to the World Health Organization 2020 guidelines on PA [12].

#### Active mobility index

The AMI assess the participant’s life space with PA and social activity. The detailed protocol has been described in a previous study [2]. The AMI assess three levels of life space in recent a month: < 1, 1–10, and > 10 km from the participant’s residence. In each area, participants were asked how often they visited the location per week (< once/1–3/4–6 days/every day); the purpose [mainly for physical activity (such as walking and exercise)/mainly for daily chores and appointments (shopping or meeting people)/and both equally]; transportation (walking/bicycle/bus, train/car, or other); extent of interaction with others [how many people (0/1–2/3–4/≥ 5)]; and extent of physical activity [how much (almost none/very little/some, or a lot)].

The life-space scores were computed for each level by multiplying the life-space level and frequency. AMI physical/social scores were computed by multiplying the life-space score and physical/social scores. Physical scores were the sum of the purpose, transportation, and extent of physical activity. Social scores were the sum of the purpose, transportation, and extent of interaction with others. The allocation of points can be found in the original literature on AMI [2]. The AMI physical and social scores ranged from 0 to 144, with higher scores indicating greater mobility with activities. Either the physical or social scores were high, or both scores were equal in response to the purpose and transportation question. The range of the AMI total score was the sum of the AMI physical and social scores from 0 to 216. The original literature provides the scoring sample in the Appendix [2].

#### Confounding factors

Data on sociodemographic characteristics (age, sex, and years of education) and medical information were collected through face-to-face interview. As the medical data, body mass index (BMI), cognitive function, depressive symptoms, fall history in a year (yes/no), whether having pain in any body part (yes/no) were assessed. Cognitive function was assessed using the MMSE [13]. Depressive symptoms were assessed using the 15-item geriatric depression scale (GDS), consisting of 15 yes/no questions and a score ranging from 0 to 15 [14]. In addition, gait speed (m/s) at a comfortable pace was measured using a stopwatch with a sensor by walking a 6.4 m path on a flat and straight surface. A 2.4 m walking path to measure walking speed was set using two markers, and 2 m sections before the start marker and beyond the end marker were set to ensure a consistent walking pace while on the timed path.

### Statistical analysis

The differences in participant’s characteristics between meeting or not meeting PA guidelines were analyzed using an independent *t*-test for continuous variables and the *χ*^2^-test for discrete variables. MVPA, LPA, and step counts among the quartile groups of AMI total, physical, and social scores were compared using one-way ANOVA and Tukey’s post hoc test. Linear associations between AMI scores and PA were examined using multiple linear regression analysis in the crude model and fully adjusted model for all covariates. Associations between the quartiles of AMI total, physical, and social scores and meeting PA guidelines were examined using binomial logistic regression models. Adjusted odds ratios (aOR) and 95% confidence intervals (CI) of quartile score groups referred to the Q1 group for meeting PA guidelines were calculated in the crude model and fully adjusted model for all covariates. To assess the discriminative ability of AMI scores to meet PA guidelines, a receiver operating characteristic (ROC) curve was used, and the area under the curve (AUC) was calculated. The optimal cut-off values of AMI scores for meeting PA guidelines were determined using the maximal Youden’s index. All analyses were performed using SPSS version 25 (IBM, New York City, NY, USA). The level of statistical significance was set at *P* < 0.05.

## Results

The characteristics of the 2499 participants (75.5 ± 4.0 years old, 54.4% female, and 12.0 ± 2.6 years of education) are presented in Table 1. The mean AMI total, physical, and social scores were 73.2 ± 29.9, 32.5 ± 17.9, and 40.7 ± 19.0, respectively. Participants had 22.0 ± 6.6 days of the valid accelerometer data, and the mean PA outputs were 25.0 ± 17.6 min/day of MVPA, 43.8 ± 17.8 min/day of LPA, and 5516 ± 2768 step counts. Among all participants, 1188 (47.5%) met the PA guidelines (MVPA ≥ 150 min/week). The meeting PA guideline group showed significantly higher AMI scores (all *P* < 0.001). The difference in the AMI physical scores between the two groups was larger than the difference in AMI social score.

**Table 1:** Participant’s characteristics

	Overall (*n* = 2499)	Meeting PA guideline^a^		*P*^b^
		No (*n* = 1311)	Yes (*n* = 1188)	
Age, years	75.5 (4.0)	76.1 (4.3)	74.9 (3.6)	< 0.001
Female, %	54.4	57.1	51.4	0.005
Education, years	12.0 (2.6)	11.9 (2.6)	12.2 (2.6)	0.037
BMI, kg/m^2^	22.8 (3.1)	23.0 (3.1)	22.6 (3)	0.001
MMSE, score	26.4 (2.3)	26.3 (2.3)	26.4 (2.2)	0.359
GDS, score	2.6 (2.6)	2.8 (2.5)	2.5 (2.6)	0.006
Fall history, %	18.8	18.8	18.8	0.997
Pain, %	48.8	51.4	45.9	0.006
Gait speed, m/s	1.11 (0.21)	1.08 (0.21)	1.15 (0.20)	< 0.001
AMI				
Total score	73.2 (29.9)	67.1 (28.4)	80.0 (30.1)	< 0.001
Physical score	32.5 (17.9)	27.3 (15.4)	38.2 (18.7)	< 0.001
Social score	40.7 (19.0)	39.8 (18.3)	41.8 (19.6)	0.007
PA				
MVPA, min/day	25.0 (17.6)	12.6 (5.3)	38.5 (16.4)	< 0.001
LPA, min/day	43.8 (17.8)	37.9 (15.1)	50.3 (18.3)	< 0.001
Step counts	5516 (2768)	3630 (1378)	7597 (2401)	< 0.001

All values are reported as mean (SD) or %

*AMI* active mobility index; *PA* physical activity; *BMI* body mass index; *MMSE* mini-mental state examination; *GDS* geriatric depression scale; *MVPA* moderate-to-vigorous-intensity physical activity; *LPA* low-intensity physical activity.

^a^Meeting PA guideline was engaging in ≥ 150 min/week of MVPA

^b^Continuous variables and category variables between groups were compared using independent *t*-test and *χ*^2^-test, respectively

**Fig. 1:**
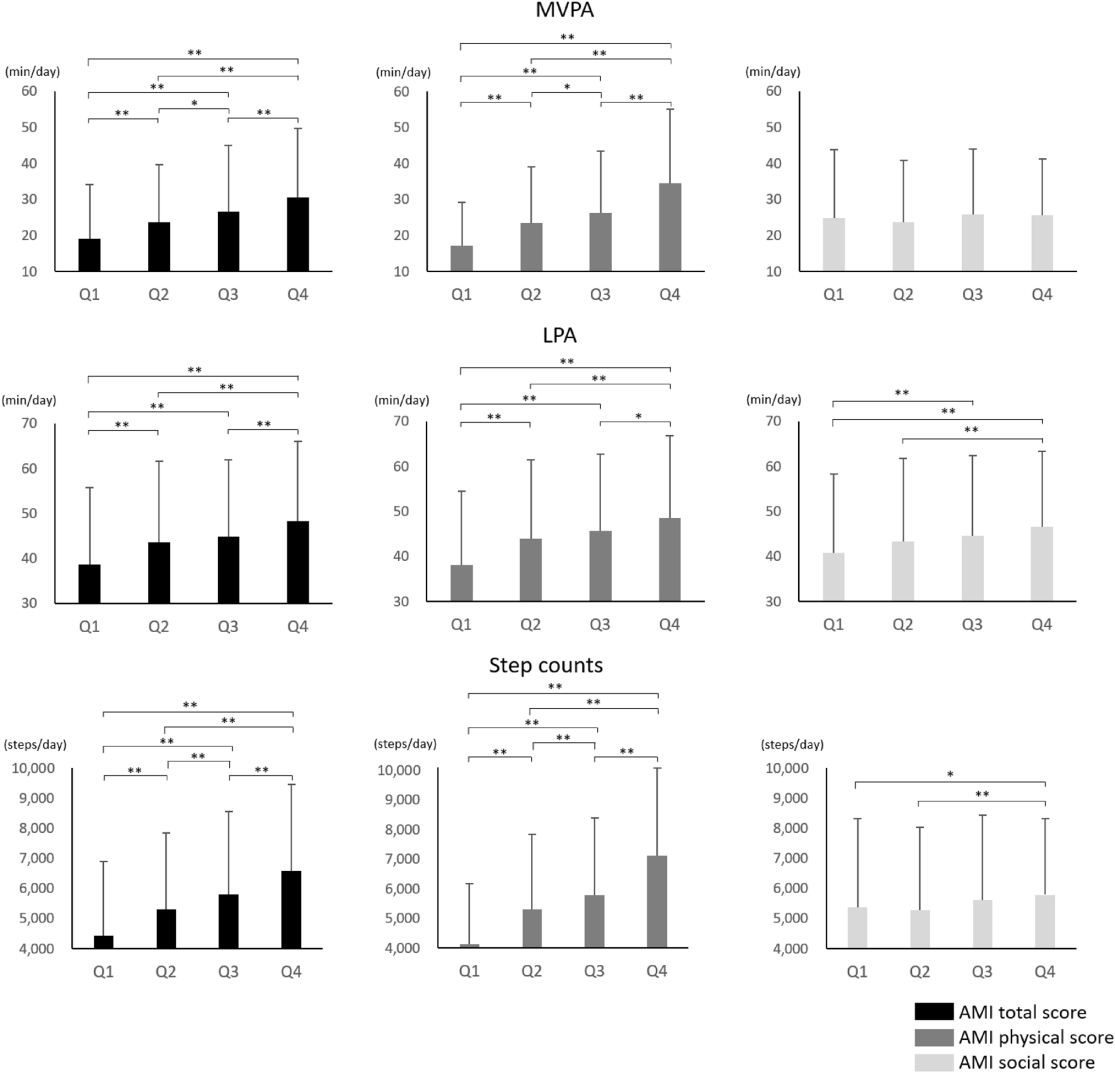
Comparison of MVPA, LPA, and step counts among quartile groups of AMI scores by one-way ANOVA and Tukey’s post hoc test. *AMI* active mobility index; *MVPA* moderate-to-vigorous-intensity physical activity; *LPA* low-intensity physical activity. *P < 0.05, **P < 0.01

The differences in PA among the quartile groups of AMI total, physical, and social scores are shown in Fig. 1. In AMI total and physical scores, higher score groups indicated longer time of MVPA and LPA and more step counts than the lower score groups. In the AMI social score, only the Q4 group showed a significantly longer time of LPA and more step counts than the Q1 and Q2 groups. The multiple linear regression models of AMI scores and PA are summarized in Table 2. AMI total and physical scores were associated with MVPA, LPA, and step counts [standardized partial regression coefficient (*β*) for MVPA, LPA, and step counts of AMI total score were 0.21, 0.16, and 0.24, respectively; all *P* < 0.001; AMI physical scores were 0.34, 0.18, and 0.36, respectively; all *P* < 0.001]. AMI social score was associated with LPA (*β *= 0.08, *P* < 0.001) and step counts (*β *= 0.04, *P* < 0.036).

**Table 2:** Multiple linear regression analyses for the associations between AMI scores and PA

	MVPA			LPA			Step counts		
	*β*	*P*	Adjusted *R*^2^	*β*	*P*	Adjusted *R*^2^	*β*	*P*	Adjusted *R*^2^
Crude model									
AMI total score	0.25	< 0.001	0.063	0.21	< 0.001	0.042	0.30	< 0.001	0.087
AMI physical score	0.38	< 0.001	0.144	0.21	< 0.001	0.045	0.41	< 0.001	0.171
AMI social score	0.04	0.069	0.001	0.12	< 0.001	0.014	0.08	< 0.001	0.005
Adjusted model^a^									
AMI total score	0.21	< 0.001	0.109	0.16	< 0.001	0.114	0.24	< 0.001	0.160
AMI physical score	0.34	< 0.001	0.174	0.18	< 0.001	0.121	0.36	< 0.001	0.224
AMI social score	0.01	0.653	0.069	0.08	< 0.001	0.096	0.04	0.036	0.108

*AMI* active mobility index; *PA* physical activity; *MVPA* moderate-to-vigorous-intensity physical activity; *LPA* low-intensity physical activity; *MMSE* mini-mental state examination; *GDS* geriatric depression scale

^a^Adjusted for age, sex, education year, body mass index, MMSE score, GDS score, fall history, pain, and gait speed

The ORs of AMI scores for meeting PA guidelines in the logistic regression model are summarized in Table 3. Higher score quartile groups of AMI total and physical scores showed a clear association with increased OR of meeting PA guidelines [aOR (95% CI): AMI total score Q2: 1.67 (1.32–2.11); Q3: 1.93 (1.52–2.45); Q4: 2.98 (2.33–3.81); AMI physical score Q2: 2.22 (1.75–2.81); Q3: 2.78 (2.19–3.54); Q4: 5.29 (4.11–6.80)]. The AMI social score was not significantly associated with meeting PA guidelines.

**Table 3:** Logistic regression model for the associations between AMI scores and meeting PA guideline

	Crude model		Adjusted model^a^	
	OR (95% CI)	*P*	OR (95% CI)	*P*
AMI total score				
Q2 (ref: Q1)	1.74 (1.38–2.19)	< 0.001	1.67 (1.32–2.11)	< 0.001
Q3	2.07 (1.65–2.60)	< 0.001	1.93 (1.52–2.45)	< 0.001
Q4	3.38 (2.67–4.26)	< 0.001	2.98 (2.33–3.81)	< 0.001
*P* for trend		< 0.001		< 0.001
AMI physical score				
Q2 (ref: Q1)	2.29 (1.82–2.88)	< 0.001	2.22 (1.75–2.81)	< 0.001
Q3	3.02 (2.39–3.81)	< 0.001	2.78 (2.19–3.54)	< 0.001
Q4	6.01 (4.72–7.65)	< 0.001	5.29 (4.11–6.80)	< 0.001
*P* for trend		< 0.001		< 0.001
AMI social score				
Q2 (ref: Q1)	0.89 (0.72–1.12)	0.320	0.84 (0.67–1.06)	0.138
Q3	1.11 (0.89–1.38)	0.356	1.06 (0.84–1.34)	0.631
Q4	1.26 (1.00–1.57)	0.047	1.11 (0.87–1.41)	0.408
*P* for trend		0.021		0.090

*AMI* active mobility index; *PA* physical activity; *OR* odds ratio; *CI* confidence interval; *BMI* body mass index; *MMSE* mini-mental state examination; *GDS* geriatric depression scale

^a^Adjusted for age, sex, education year, BMI, MMSE score, GDS score, fall history, pain, and gait speed

The AMI physical score had the highest predictive ability for identifying meeting PA guidelines among the AMI scores (AUC 0.68, 95% CI 0.66–0.70). The cut-off value of the AMI physical score was 35, with 52.9% sensitivity and 72.9% specificity. The AUC of AMI total score was 0.63 (95% CI 0.61–0.65, cut-off 66, sensitivity 67.8%, specificity 50.8%), and that of AMI social score was 0.53 (95% CI 0.51–0.55, cut-off 40, sensitivity 52.2%, specificity 53.3%).

## Discussion

The present study examined the association between AMI scores and objectively measured PA among community-dwelling older adults. Higher AMI total and physical scores were associated with longer time of MVPA, LPA, and more step counts, and with increased OR for meeting PA guidelines. The AMI social score showed a weak association with LPA. The AMI physical score had the highest predictive ability among the AMI scores to identify those who met the PA guidelines.

Our findings showed that higher AMI total and physical scores were associated with higher PA. This was consistent with previous studies that showed that a larger life space was associated with a more objectively measured PA [6, 7]. AMI total and physical scores considered the extent of PA, which probably led to the association between MVPA and meeting PA guidelines. Although the AUC value was not high for AMI physical scores to discriminate meeting PA guidelines, older adults with higher AMI physical scores are likely to meet the recommended PA guidelines. Therefore, future study is required to refer these findings when examining the association between AMI physical score and health outcomes.

On the other hand, the higher AMI social score group engaged in longer periods of LPA, and the AMI social score had a weak association with MVPA and step counts. Other studies support the finding of an association between AMI social score and LPA. Social isolation was related to reduced LPA in older adults [15], and social connectedness moderated the negative association between loneliness and self-reported PA [16]. Therefore, older adults with higher AMI social scores might engage in more LPA. Systematic reviews have reported that engaging in LPA has health benefits, including cardiometabolic health and mental health [17–19], and in a cohort study, older adults with higher AMI social scores were associated with reduced OR of depressive symptoms and physical frailty [2]. Thus, the AMI social score may be associated with health outcomes not only through social activity but also through LPA.

The strength of the present study is that it examined the associations between AMI and PA using large cohort data, and the findings from this study add to the evidence concerning the association between life space and PA [6, 7]. Our study had some limitations. First, it had a cross-sectional design and could not examine the causal relationships between AMI and PA. Future studies with longitudinal designs are needed to examine causal relationships. Second, there was selection bias because the participants were not randomly selected. They were relatively healthy and able to access examination places from their homes, which might distort the association between AMI and PA among community-dwelling older adults. Third, because participants wore the accelerometer on the waist and could not wear it during water-based activities such as swimming, there was a possibility that we could not assess PA by upper limbs or water-based activity.

## Conclusions

Higher AMI scores were associated with a higher PA. In particular, older adults with higher AMI total and physical scores engaged in longer MVPA, LPA, and more step counts. Future studies can refer to the present findings when estimating PA in older adults from AMI scores and examining the association between AMI scores and health outcomes.

## Data Availability

The datasets used and/or analyzed during the present study are available from the corresponding author on reasonable request.
